# Anatomical Joint Form Variation in Sacroiliac Joint Disease: Current Concepts and New Perspectives

**DOI:** 10.1007/s11926-021-01033-7

**Published:** 2021-07-03

**Authors:** Katharina Ziegeler, Kay Geert A. Hermann, Torsten Diekhoff

**Affiliations:** Department of Radiology, Charité-Universitätsmedizin Berlin, Charitéplatz 1, 10117 Berlin, Germany

**Keywords:** Sacroiliac joint, Anatomical variation, Axial spondyloarthritis

## Abstract

**Purpose of Review:**

The aim of this article is to further the understanding of anatomical variation of the sacroiliac joint (SIJ) within the rheumatological community and point out promising fields of research in the interplay of SIJ anatomy and joint disease.

**Recent Findings:**

Mechanical strain has long been implicated in onset and progression of axial spondyloarthritis (axSpA). Recent investigations found changes in the pattern of degenerative lesions of the SIJ in the normal population in patients with atypical joint forms. Furthermore, atypical SIJ forms are more prevalent in patients with axial spondyloarthritis and mechanical SIJ disease.

**Summary:**

Mechanical stress from anatomical joint form variation may have an impact on development and progression of axSpA. Furthermore, mechanically induced bone marrow edema may act as an axSpA mimic on MRI and needs to be more accurately classified.

## Introduction

The question of mechanical factors in the development and progression of axial spondyloarthritis (axSpA) is of great interest to the scientific community and inspires ongoing discussion among experts in the field [1, 2]. Within this area of research, a special focus lies on the sacroiliac joints (SIJ), which biophysically act as the main transductor of force from the lower extremities to the torso [3] and are also by far the most common site of manifestation of axSpA [4]. Recent studies have found associations between the shape of the joints themselves and both inflammatory and degenerative joint disease [5•]. The aim of this article is to give a brief overview of current concepts of the interplay of mechanics and inflammation at the SIJ in axSpA, to further the understanding of anatomical joint form variation within the rheumatological community and point out promising fields of research in the interplay of SIJ anatomy and joint disease.

## Mechanical Factors of Inflammation

Mechanical strain has long been hypothesized to play a role in inflammatory arthritis—a concept that has gained increasing traction within the scientific community in recent years [6••]. In axSpA, mechanically induced micro-trauma at the enthesis is assumed to trigger aberrant repair in the presence of IL-17-IL23-mediated immunity, resulting in ectopic bone growth [7]. Cambré et al. investigated the effect of mechanical stress and unloading in collagen-induced arthritis (CIA) and TNF overexpression (TNFdARE) mouse models and found that excess mechanical load accelerated the onset of arthritis and its persistence [8, 9]. Jacques et al. showed that mechanical unloading inhibits the development of new bone formation and that mechanical stress promotes pro-inflammatory pathways in a model of CIA induced in DBA/1 mice [10].

Clinically, mechanical stress has been implicated in the onset of both rheumatoid arthritis [11] and psoriatic arthritis [12]. Although the effect of exercise is widely accepted to be beneficial for axSpA patients due to its immune-modulatory effect [13, 14], there have long been reports of increased disease activity in individuals exposed to mechanical stress. Ward et al. showed that physically demanding occupations were associated with more functional limitations than sedentary ones [15]. Recent clinical studies by Bindesboll et al. [16] and Bakirci et al. [17] found more spinal stiffness, more new bone formation, and higher BASDAI scores in obese axSpA patients and attributed these at least in part to the increased axial load in overweight individuals.

## Biomechanics and Typical Anatomy of the Sacroiliac Joints

The SIJ complex is one of the most important mechanical axes in the human body, as force from the lower extremities is translated to the lumbar spine mainly through this joint [18]. The wedge shape of the sacrum as well as many small groves and ridges on the joint surface provides structural and frictional stability against axial loading [3]. Physiologically, the SIJ has a very limited range of motion (a tilt-like movement of the sacrum against the pelvic ring called nutation) which is further restricted by a very firm capsule, strong stabilizing ligaments, and muscles of the pelvic floor (levator ani and coccygeus) [18]. The SIJ, as part of the pelvic ring, exhibits significant sexual dimorphism: the male sacrum is generally narrower, more even, and more curved than the female [3]. The male sacral cartilage is typically thinner while the overall joint surface area is greater in males [18]. Biomechanically, the main role of the SIJ is that of a shock absorber, especially during bipedal walking [19]. A recent study by Joukar et al. investigated differences in joint biomechanics between males and females [20] in an elaborate computational finite element model of the SIJ—they found higher mobility, mechanical stresses and loads, and ligamentous strains of the female joint.

## Classification of Sacroiliac Joint Form Variants

**Fig. 1:**
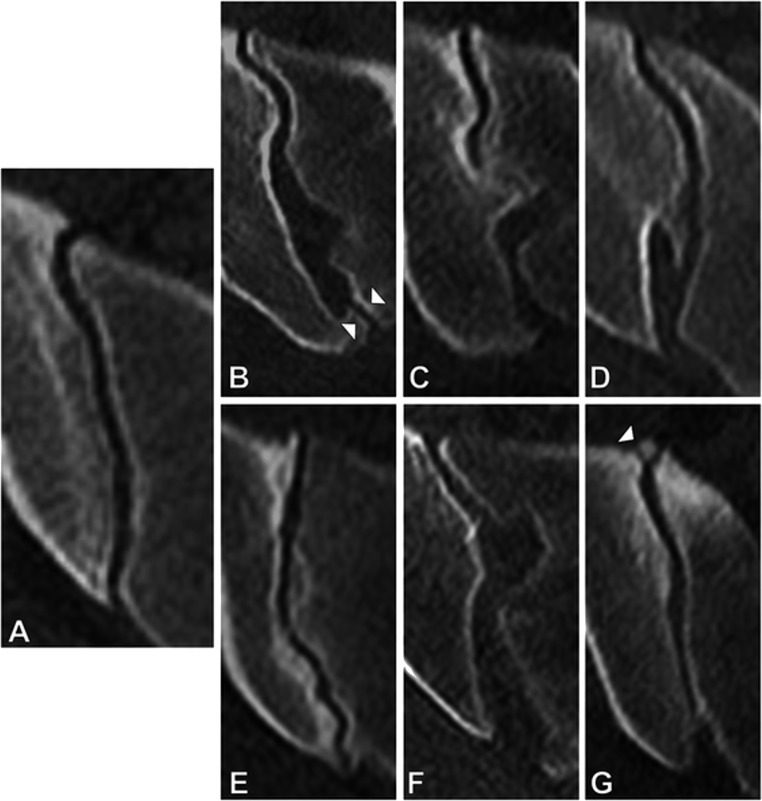
Joint forms. According to the classification by Prassopoulos et al. [21]. Axial reconstructions of computed tomography images. (A) Typical joint. (B) Accessory joint (indicated by white arrowheads). (C) Iliosacral complex. (D) Bipartite ilium. (E) Crescent-shaped ilium. (F) Semi-circular defects. (G) Sacral ossification center (indicated by white arrowhead). Images adapted with the authors’ permission from [22]

The categorization of joint forms on cross-sectional imaging was pioneered by Prassopoulos et al. more than 20 years ago [21]. They proposed 6 distinct form variations of the typical SIJ (see Fig. 1). Among these, the accessory SIJ (Fig. 1B) is likely the one most familiar to most clinicians—this term is used, whenever an additional joint facet is observed dorsally to the joint proper. Accessory joints are associated with chronic degenerative lesions of the SIJ [22]: patients with this specific joint form had an odd’s ratio of 2.7 for sclerosis in the dorsal joint portion in a large cross-sectional study of SIJ CT from the normal population [22]. Furthermore, they have been described as possible causes of low back pain in case reports [23, 24]. The most common joint form anomaly in the general population is the bipartite ilium (Fig. 1D) [22], which is a term used for non-union of two iliac bony plates, resulting in a configuration of the ilium which resembles a crab claw. This joint form is very rare in men (0.7%) but extremely common in women (21.9%). In a study on joint form frequencies in patients with axSpA, mechanical joint disease (osteoarthritis, osteitis condensans ilii), and healthy controls [5•], we found comparatively high frequencies of both the iliosacral complex (Fig. 1C) and the crescent-shaped ilium (Fig. 1E). These two joint forms may be interpreted as two ends of a spectrum: form a very convex configuration of the ilium against the sacrum with a protruding nudge to an overall concave ilium; the centre of this spectrum would then be the typical joint with its only slightly convex shape.

In our clinical experience, classification into these categories may be less than unequivocal, even among experts in the field—further studies on the reproducibility of joint form assessment by rheumatologists and radiologists are warranted. To date, it is also unclear whether the six described joint forms capture all and also the relevant variations that result in clinical symptoms and the development of a disease. A refinement of the original system and a re-evaluation of the different forms in respect of their impact on clinical symptoms, imaging findings, and the development of a disease are needed. Additionally, a quantitative approach to joint form assessment may be more appropriate to further the understanding of changes in biomechanical load and possible mechanical conflicts.

## Joint Forms and SIJ Disease

**Fig. 2:**
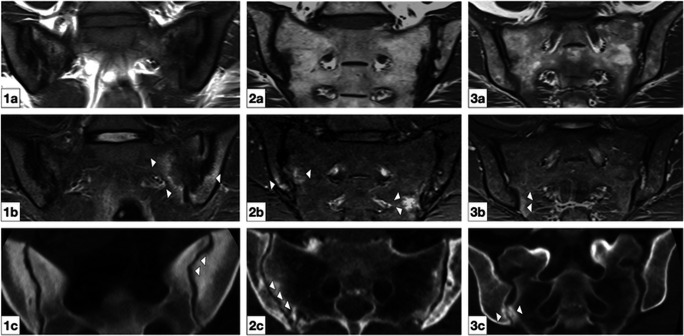
Examples of joint lesions in atypical joints. Row a: T1 weighted oblique coronal MR images. Row b: short-tau inversion recovery (STIR) oblique coronal MR images. Row c: oblique coronal CT reconstructions. Patient 1 (column 1): female patient with osteitis condensans: note the marked sclerosis, the ilium notch, or iliosacral complex (panel 1c, arrowheads) as well as the bone marrow edema on STIR imaging (panel 1b, arrowheads). Patient 2 (column 2): female with osteoarthritis of the SIJ: note the overall concave shape of the ilium (panel 2c, arrowheads) and the marked bone marrow edema adjacent to the joint on STIR imaging (panel 2b, white arrowheads). Patient 3: female with osteitis condensans. An accessory joint is seen almost exclusively on CT (panel 3c, arrowheads), but a faint bone marrow edema can be seen in STIR imaging (panel 3b, arrowhead)

To date, the evidence base of the association of sacroiliac joint disease and joint form variation is still sparse. In a large study on more than 800 patients without SIJ-related symptoms, we found a significant association between joint sclerosis and accessory joint facets (OR 2.7) [22]—the observed sclerosis is typically found in the dorsal and caudal aspects of the joints, indicating a pathological bony contact (see Fig. 2). In joints with an iliosacral complex, sclerosis in the ventral and dorsal joint aspect is less common, but the risk of ventral osteophytes is increased more than threefold (OR 3.6) [22]. These findings indicate that different joint shapes have a significant impact on load distribution within the joint, altering the natural course of degeneration.

We furthermore investigated the proportion of atypical joints in axSpA patients compared to both healthy controls and patients with mechanical joint disease (osteoarthritis, osteitis condensans) [5•]. In this study, we found a high propensity for atypical joint forms in patients with mechanical joint disease (80.3%) compared to axSpA patients (44.1%) and controls (37.5%). Atypical joint forms were significantly more prevalent in males with axSpA (32.2%) than in healthy controls (13.9%)—the most common atypical forms in this group were the iliosacral complex (12.9%) and the crescent-shaped ilium (6.5%) [5•]. As these shapes are rare in patients without joint disease [22], their role in disease mechanisms deserves further attention within the scientific community.

An important aspect of joint form variation and sacroiliac joint disease is the potential of mechanically induced bone marrow edema to mimic inflammatory osteitis adjacent to the joint. Mechanical strain as a mimic of inflammation has been studied in various setting in previous years [25•]. Varkas et al. found bone marrow edema of the SIJ in 50.0% of military recruits exposed to intense physical strain during training [26], and Weber et al. found bone marrow edema in 30–35% of runners and 41% of ice-hockey players [27]. Furthermore, Eshed et al. [28] and Agten et al. [29] found a high prevalence of bone marrow edema reminiscent of sacroiliitis in healthy pregnant and post-partum women. El Rafei et al. [30] investigated MRI joint lesions in patients with atypical joint forms; they found structural and edematous changes in 18% of patients with accessory joints. As patients fulfilling the ASAS criteria for active inflammation were excluded from this analysis, however, individuals with both active inflammation and mechanical bone marrow edema form joint form variation were not studied.

## Imaging of Joint Forms

The orientation and form of the SIJ in three-dimensional space is surprisingly complex. Therefore, the reliable assessment of the form variations needs three-dimensional imaging with computed tomography. This is also the imaging modality, for which most studies have been done and on which the classification of the different joint forms relies. While MRI also generates cross-sectional images, the typical oblique-coronal orientation, the rather thick slices (3–5 mm compared to < 1 mm in CT), and the limited coverage of the image stack present a challenge for proper assessment. However, the assessment of the impact of atypical joints on mechanical reactions in form of edema or other bone marrow lesions remains the domain of MRI. Therefore, the optimal diagnostic approach to anatomical variation–induced stress consists of a CT scan to detect whether an anatomical variation is present and an MRI to assess the pathophysiological stress reactions of the bone, mainly in the form of bone marrow edema. However, recent developments in imaging techniques such as dual-energy CT (for detection of bone marrow lesions) [31, 32] or direct depiction of the bone in MRI, e.g., with synthetic CT [33], have the potential to improve the diagnostic workup. So far, classification of sacroiliac joint form in radiography has not been attempted. Therefore, a reliable detection and evaluation of the impact in an individual patient remains the domain of MRI and CT.

## Impact on Clinical Decision-making

In our own clinical practice, we have encountered a number of patients with clinical suspicion of axSpA, in whom further imaging revealed that the observed bone marrow edema can be attributed to joint form variation (see Fig. 2). In so far, those conditions can be valid differential diagnoses for an inflammatory disease as they may mimic axSpA in clinical symptoms and certain imaging characteristics such as bone marrow edema, sclerosis, erosions, or even partial ankylosis that result from mechanical stress in the absence of a pathological inflammatory reaction. On the other hand, our first data show that form variations are also abundant in an axSpA collective, suggesting either a misclassification or—and more likely—the triggering or maintenance of the inflammation from persistent mechanical stress. Therefore, as of now, the presence of an atypical joint form does not exclude axSpA or related diagnoses. However, it can serve as a mechanical explanation in clinical circumstances, where a rheumatic disease seems unlikely.

## Conclusion

Many of the findings discussed in this review stem from exploratory analyses that require verification in larger patient cohorts. Most importantly, reliable data on the MRI findings of atypical joints in axSpA patients are still lacking. Furthermore, the impact of mechanical stress from joint form variation may represent an interesting field of enquiry in the research of residual disease activity in long-term observational studies of axSpA. The evidence of biomechanical stress from atypical joint forms is derived from the observation of degenerative lesions, while direct proof of differences in load distribution, e.g., from computational biophysical models, is still lacking. Lastly, higher mobility and stresses of the female SIJ shown in computational models as well as its propensity to exhibit joint form variation beg the question, why females are less inflicted by inflammatory diseases of the SIJ—further research on gender aspects is warranted.

In summary, mechanical stress derived from joint form variation represents a promising field of research to further the understanding of the interplay of mechanical factors and inflammation in axSpA. A detailed understanding of frequency, extent, and spatial distribution of both structural lesions and bone marrow edema observed in patients with variant joint forms is essential to avoid overdiagnosis of axSpA in atypical joints.
